# Opioid biomarkers in urine as reliable and valid correlates of opium use characteristics: A 10-year longitudinal assessment

**DOI:** 10.1016/j.dadr.2025.100377

**Published:** 2025-08-30

**Authors:** Mahdokht Naghash, Rebecca L. Shaner, Hossein Poustchi, Gholamreza Roshandel, Katrice D. Williams, Abraham Tuachi, Farin Kamangar, Paolo Boffetta, Christian C. Abnet, Elizabeth I. Hamelin, Neal D. Freedman, Reza Malekzadeh, Arash Etemadi

**Affiliations:** a Digestive Oncology Research Center, Digestive Diseases Research Institute, Tehran University of Medical Sciences, Tehran, Iran; b Emergency Response Branch, Division of Laboratory Sciences, National Center for Environmental Health, Centers for Disease Control and Prevention, Atlanta, GA, United States; c Liver and Pancreaticobiliary Research Center, Digestive Diseases Research Institute, Tehran University of Medical Sciences, Tehran, Iran; d Golestan Research Center of Gastroenterology and Hepatology, Golestan University of Medical Sciences, Gorgan, Iran; e Battelle Memorial Institute at the Centers for Disease Control and Prevention, Atlanta, GA, United States; f Department of Biology, School of Computer, Mathematical, and Natural Sciences, Morgan State University, Baltimore, MD, United States; g Stony Brook Cancer Center, Stony Brook University, Stony Brook, New York, United States; h Department of Medical and Surgical Sciences, University of Bologna, Bologna, Italy; i Metabolic Epidemiology Branch, Division of Cancer Epidemiology and Genetics, National Cancer Institute, NIH, Bethesda, MD, United States; j Tobacco Control Research Branch, Division of Cancer Control and Population Sciences, National Cancer Institute, NIH, Bethesda, MD, United States

**Keywords:** Opium, Opioid biomarkers, Self-reports, Opioid use disorder

## Abstract

**Background:**

Biomarkers can clarify the mechanistic bases of health effects associated with opiate use and improve evaluating dose-response relationships by quantifying the absorbed dose through different routes and patterns of use, supporting the generalizability of opium research findings to broader opioid use.

**Methods:**

We recruited 449 individuals who used opium and 66 individuals who did not, 10 years after baseline evaluation in a cohort study. At both time points, we collected self-reported characteristics of opium use (route, frequency, type, and dose) and measured urinary concentrations of codeine, hydrocodone, hydromorphone, morphine, morphine-3-glucuronide, and morphine-6-glucuronide in spot urine samples. We used multivariate linear regression models to determine the independent effects of each opium use characteristic on biomarker concentrations. Reliability of biomarker concentrations over the 10-year interval was assessed using intraclass correlation coefficients (ICCs) from linear mixed-effect models.

**Results:**

At the follow-up, 229 (51.0 %) subjects used opium by ingestion, which showed a significant shift compared with baseline (24.4 % ingestion). In adjusted models, opium ingestion, daily use, and presence of opioid use disorder (OUD) were associated with higher concentrations of all opioid biomarkers compared with opium smoking, non-daily use, and absence of OUD, respectively. All opioid biomarkers showed significant dose-response relationships relative to self-reported doses. Biomarker concentrations peaked when opium was used 3–4 h before sample collection and declined afterwards, remaining detectable for several days. Biomarker measurements were reliable (ICCs between 0.69 and 0.78) over the 10-year interval.

**Conclusions:**

Opioid biomarkers are valid markers of lifetime history, route, frequency, dose, and recency of opium use and OUD diagnosis, and demonstrate good long-term reliability.

## Highlights

• Opioid biomarkers were correlated with route, dose, frequency, and recency of use.
• Confirmed opioid use disorder was associated with higher opioid biomarker levels.
• Opioid biomarker levels remained detectable for days after opium use.
• Opioid biomarker levels showed good reliability during a 10-year interval.

## 1 Introduction

According to the 2024 World Drug Report, opioids are the second most used drugs globally, and opioid use has the highest impact on the global burden of disease compared with other substances. In this report, 59.5 million people used non-medical opioids globally, half of whom used opiates, the natural derivatives of *Papaver somniferum* (UNODC, 2024). Among these, opium, used regularly by over 5 million people (Rahimi-Movaghar et al.,2018), is notable for its wide range of health impacts. Opium consumption has been recognized as carcinogenic to humans by the International Agency for Research on Cancer (IARC) and is associated with an increased risk of mortality and cardiovascular diseases, among others (Bijani et al., 2022, IARC Working Group on the Identification of Carcinogenic Hazards to Humans, 2021, Khademi et al., 2012, Malekzadeh et al., 2013, Najafipour and Beik, 2016, Nakhaee et al., 2020, Nalini et al., 2021). In high-consumption countries, opium is sold as a street drug with varying amounts of adulterants and is used in different forms (e.g., raw or refined) and by various routes (e.g., smoking or ingestion) (IARC Working Group on the Identification of Carcinogenic Hazards to Humans, 2021). These variations complicate opium research and impede the generalizability of its findings to the broader context of prescription and illicit opioid use.

Current methods for assessing opium use in epidemiological studies predominantly rely on self-reported data (IARC Working Group on the Identification of Carcinogenic Hazards to Humans, 2021), which are prone to bias and may not capture the details of use as effectively as biological markers (Khalili et al., 2021, Rashidian et al., 2017, Rendon et al., 2017). While self-reports have been shown to be reliable measures of the broad history of lifetime “ever” opium use (Abnet et al., 2004), their accuracy in reflecting detailed characteristics of use, such as frequency, route, and type of use, is unknown. Furthermore, inferring a causal role for opium in disease outcomes requires identifying dose-response relationships that quantify the observed associations and providing direct mechanistic evidence through measuring the absorbed dose as a proportion of the consumed dose (Torres, 2015). Such assessments for the incidence of multiple cancers and gastrointestinal disease mortality have solely depended on the perceived weight of opium products, which may be unreliable and underestimated (Malekzadeh et al., 2013, Mohebbi et al., 2019, Sheikh et al., 2020). Validating subjective characterizations through biomarker panels can improve the robustness of opium research, facilitate evaluating dose-response relationships, and clarify the mechanistic evidence associated with the internal absorbed doses through different routes and patterns of use.

Opium is comprised of several alkaloids, among which morphine and codeine are the most abundant (Labanca et al., 2018, Lim and Kwok, 1981). Approximately 12–16 % of the dried weight of opium is attributed to morphine and 2–3 % to codeine (Lim and Kwok, 1981). These alkaloids undergo extensive hepatic metabolism, producing various metabolites that are ultimately excreted in the urine alongside a small amount of unchanged alkaloids (Dinis-Oliveira, 2019, Hasselström and Säwe, 1993, Smith, 2009, Williams et al., 2001). Morphine is predominantly metabolized into morphine-3-glucuronide (60 %), followed by morphine-6-glucuronide (5–10 %) and hydromorphone (0.02–12 %). Codeine is metabolized into morphine (0–15 %) and hydrocodone (11 %), with hydrocodone further undergoing partial metabolism to hydromorphone (Dinis-Oliveira, 2019, Hutchinson et al., 2004, McDonough et al., 2008, Oyler et al., 2000, Smith, 2009, Thorn et al., 2009). Excretion of these opioids in the urine following opium use has been observed for up to 2–4 days after use (Abnet et al., 2004, Hasselström and Säwe, 1993, Smith, 2009, Yaksh and Wallace, 2017).

To address the presented gaps, we conducted a study nested within the Golestan Cohort Study (GCS), which has successfully followed over 8400 individuals who used opium in Golestan Province, Iran (Pourshams et al., 2010). GCS data includes comprehensive opium use histories, comprising route, type, frequency, and dose of usage, making it a uniquely suitable sample for opium research. We used self-reported opium use histories and urine samples collected from 543 of the GCS participants at baseline and after 10 years. Urine samples were analyzed for 6 opioid biomarkers, consisting of two opium alkaloids and their metabolites. We evaluated the consistency of these biomarkers over a decade, examined how changes in use patterns correlated with biomarker concentrations, and compared concentrations across different routes, types, frequencies, intensities, and intervals since the last use. This analysis aimed to correlate self-reported characteristics of opium use with opioid biomarker concentrations and evaluate the consistency of opioid biomarker concentrations as markers of use across an interval of 10 years.

## 2 Methods

### 2.1 Study population

The GCS recruited 50,045 participants from the general population of Golestan Province in northeastern Iran between 2004 and 2008 (Pourshams et al., 2010). At baseline, comprehensive opium and tobacco use questionnaire data and spot urine samples were collected from all participants, including 8486 individuals who used opium. In 2017 (about 10 years after the baseline), as a nested study within the cohort, 543 GCS participants (including 451 individuals who self-reported opium use at baseline and 92 individuals who never used opium) were recalled for a follow-up session involving repeated questionnaire data and urine sample collection. These individuals were selected through stratified random sampling based on sex, tobacco use, and route and frequency of opium use, with proportions reflecting their prevalence among all GCS participants.

All participants provided written informed consent. The GCS has been approved by the ethics committees of the Tehran University of Medical Sciences and the National Cancer Institute (NCI). The analysis of de-identified samples at the Centers for Disease Control and Prevention (CDC) laboratory did not constitute human subject research.

### 2.2 Data collection

Trained interviewers administered the opium use questionnaire, which included questions on the initiation and cessation ages, type of opium, route, dose, and frequency of use, and the time since last use. This questionnaire has been previously validated against urinary codeine and morphine concentrations with a sensitivity of 93 % and specificity of 89 % for self-reported ever use (Abnet et al., 2004). We also used the validated L section of the Farsi translation of the Composite International Diagnostic Interview (CIDI 2.1-lifetime version) and Diagnostic and Statistical Manual of Mental Disorders, Fifth Edition (DSM-5) to diagnose opioid use disorder (OUD), details of which have been published before (Alvand et al., 2023).

The type of opium used was either teriak (raw opium) or shireh (a refined product made by boiling opium dross in water, with or without teriak), and the route of use was either ingestion or smoking. Recency was defined as the hours since the last use before urine sample collection, categorized into quartiles. Opium use was classified as daily or non-daily based on the frequency of use per week. Intensity of use was the consumption dose in self-reported weekly grams, which was also categorized into quartiles.

### 2.3 Laboratory measurements

The urine samples of both time points were stored in the NCI Frederick Central Repository, Fisher BioServices, at −20°C with tight freezer temperature control, ensuring adequate stability of opioid biomarkers. Samples were tested for urinary codeine, hydrocodone, hydromorphone, morphine, morphine-3-glucuronide, and morphine-6-glucuronide by the Emergency Response Branch of the Division of Laboratory Sciences (DLS), National Center for Environmental Health, CDC.

The biomarkers were measured using highly sensitive liquid chromatography tandem mass spectrometry (LC-MS/MS) assays. Analytes were measured using a SCIEX 6500 QQQ MS (Framingham, MA, USA) in positive ion mode. Two transitions were monitored for each analyte, and one transition was monitored for each isotopically labeled internal standard. Analytes and internal standards were optimized via manual infusion with the specific parameters listed in Supplementary Table S1. A calibration curve was constructed using nine calibrators with matched internal standard. Linear regression analysis of the calibrator concentration versus the ratio of the quantification ion area to the internal standard ion area was used to determine the calibration curves with a 1/x2 weighting applied. Only calibration curves with a correlation coefficient of 0.990 or greater were accepted for use.

Pooled urine lots were purchased from Tennessee Blood Services (Memphis, TN, USA). Quality control (QC) materials were also prepared in pooled urine at spiked concentration of 800, 3500, and 9000 ng/mL for codeine and morphine; 80.0, 350, and 900 ng/mL for hydrocodone, 15.0, 85.0, and 200 ng/mL for hydromorphone; and 2000, 6000, and 12.000 ng/mL for morphine-3 glucuronide and morphine-6 glucuronide. A blank QC was also made for all analytes. The analytical method was characterized by assessing 20 sets of QC samples over the course of two weeks with a minimum of two analysts. Method accuracy was determined to range from 93.6 % to 107 % and the coefficient of variation (CV) ranged from 2.52 % to 8.28 % for the QC samples (Supplementary Table S2).

To expand the upper reportable range, a protocol for dilution was developed. This protocol diluted 10 µL of the sample with 990 μL of blank urine prior to the sample preparation described above. This dilution was tested, and the accuracy was within 10 % of the spiked value for all compounds except hydrocodone. This updated the reportable ranges to the following: codeine and morphine 125–1500,000 ng/mL, hydromorphone 5.00–30,000 ng/mL, morphine-3 glucuronide and morphine-6 glucuronide 250–2000,000 ng/mL, and hydrocodone remaining 15.0–1500 ng/mL. Among those who used opium, opioid biomarker concentrations below LRL (lower reportable limit) had a prevalence of 5–25 % and were replaced by the LRL divided by the square root of 2. Those above URLs (upper reportable limit) comprised 0–5 % of all values and were imputed with 1.7 times the URL (Hornung and Reed, 1990, Sanford et al., 1993).

### 2.4 Statistical analysis

Participants who used opium at baseline but reported having never used opium during the follow-up visit (N = 12) were excluded. Those initially considered to not have used opium at baseline who, during the follow-up, reported having initiated opium use before the enrollment date (N = 10) were reclassified into the group of those who used opium. Among the remaining 82 people who did not report opium use, most (N = 66) had opioid biomarker concentrations below the LRL. However, we observed discordant concentrations in 16 of those who did not use opium, with some biomarkers below the LRL and others unexpectedly high. These individuals who did not use opium were excluded due to the fact that they seemed to have some exposure to opiates. The final analytical sample included 449 individuals who used opium at follow-up and 66 confirmed as not having used opium at all.

Demographic information was extracted from the baseline questionnaire. Descriptive statistics are reported as numbers with frequencies for categorical variables and means ± standard deviations or medians and interquartile ranges for continuous variables. Differences were evaluated using chi-squared tests, t-tests, and Wilcoxon rank-sum tests, as appropriate. Changes in the route of opium use were assessed using McNemar's test. Opioid biomarker concentrations were log-transformed to address skewed distribution and corrected for urinary creatinine to account for urinary dilution (Taracha et al., 2005), and reported as geometric means and 95 % confidence intervals (95 % CI). To allow for comparison with previous research, we also reported the geometric means of non-corrected concentrations. We used multivariate linear regression models with each biomarker as a dependent variable and opium type, route, frequency, and intensity as independent variables to determine the effect of each variable on biomarker concentration adjusted for the other use characteristics. Models for the follow-up data also included recency of use. To explore how the changes in the patterns of opium use (e.g., smoking to ingestion) impact the opioid biomarker concentrations, linear regression models were applied to follow-up data with adjustments for sex, age, recency of use, changes in the intensity of use, and the baseline concentration of the same opioid biomarker. The reliability of opioid biomarker measurements over the study period was assessed using linear mixed-effects models, with two random intercepts to account for between-participant and within-participant variability. We report intraclass correlation coefficients (ICCs), as measures of consistency over time, from these linear mixed-effects models.

All analyses were done with the Stata software version 18.0 (StataCorp Inc., College Station, TX, USA), with statistical significance set at P < 0.05. Data visualizations were created using R software version 4.3.1 (R Core Team, Vienna, Austria).

## 3 Results

The demographic and opium use characteristics of the participants are summarized in Table 1. Among 449 who used opium in the follow-up visit, 229 used opium by ingestion, 184 by smoking, and 9 by both routes (data on route of use was unavailable for 27 individuals). Those who used opium, particularly those who ingested opium, were older than those who did not use opium and more likely to reside in rural areas, lack formal education, and use tobacco. Opium use, especially smoking, was more prevalent among men. The prevalence of OUD among individuals who reported opium use was 57.9 %, with higher rates associated with ingestion compared to smoking. Ingestion of opium was also associated with increased frequency of daily use, higher intensity, and shorter recency of use compared with smoking.

**Table 1 –:** Demographic and opium use characteristics of the participants, stratified by follow-up state and route of opium use.

		**Follow-up route of opium use**				
		**Ingestion (n = 229)**	**Smoking (n = 184)**	**Dual** **(n = 9)**	**Any use (n = 449)**⁎	**No use (n = 66)**
**Age: mean (SD)**		51 (6.5)^a^, ^b^	49 (6.5)	53 (8.5)	50 (6.6)^a^	48 (6.2)
**Sex: n (%)**	**Female**	51 (22.3)	12 (6.5)	4 (44.4)	68 (15.1)	20 (30.3)
	**Male**	178 (77.7)^a^, ^b^	172 (93.5)^a^	5 (55.6)	381 (84.9)^a^	46 (69.7)
**Ethnicity: n (%)**	**Turkmen**	176 (76.9)	105 (57.1)	8 (88.9)	306 (68.2)	39 (59.1)
	**Non-Turkmen**	53 (23.1)^a^, ^b^	79 (42.9)	1 (11.1)	143 (31.8)	27 (40.9)
**Residence: n (%)**	**Urban**	83 (36.2)	106 (57.6)	4 (44.4)	209 (46.5)	66 (100)
	**Rural**	146 (63.8)^a^, ^b^	78 (42.4)^a^	5 (55.6)	240 (53.5)^a^	0 (0)
**Formal education: n (%)**	**None**	115 (50.2)	40 (21.7)	5 (55.6)	166 (37.0)	11 (16.7)
	**Any**	114 (49.8)^a^, ^b^	144 (78.3)	4 (44.4)	283 (63.0)^a^	55 (83.3)
**Tobacco use: n (%)**	**Baseline**	160 (69.9)^a^, ^b^	102 (55.4)^a^	3 (33.3)	281 (62.6)^a^	14 (21.2)
	**Follow-up**	144 (62.9)^a^, ^b^	83 (45.1)^a^	3 (33.3)	244 (54.3)^a^	15 (22.7)
**Opioid use disorder: n (%)**		152 (66.4)^b^	87 (47.3)	3 (33.3)	260 (57.9)	
**Baseline route of use**^c^**: n (%)**	**Ingestion**	99 (43.4)	0 (0)	9 (100)	109 (24.4)	
	**Smoking**	119 (52.2)^b^	183 (100)	0 (0)	327 (73.2)	
	**Dual**	10 (4.4)	0 (0)	0 (0)	11 (2.5)	
**Baseline opium type**^c^**: n (%)**	**Teriak**	185 (81.1)	170 (92.9)	7 (77.8)	386 (86.4)	
	**Shireh**	37 (16.2)	11 (6)	2 (22.2)	52 (11.6)	
	**Dual**	6 (2.6)^b^	2 (1.1)	0 (0)	9 (2.0)	
**Follow-up opium type**^d^**: n (%)**	**Teriak**	89 (40.8)	106 (57.9)	2 (22.2)	198 (48.2)	
	**Shireh**	126 (57.8)	71 (38.8)	6 (66.7)	203 (49.4)	
	**Dual**	3 (1.4)^b^	6 (3.3)	1 (11.1)	10 (2.4)	
**Baseline frequency of use**^c^**: n (%)**	**Non-daily**	41 (18)	101 (55.2)	0 (0)	159 (35.6)	
	**Daily**	187 (82)^b^	82 (44.8)	9 (100)	288 (64.4)	
**Follow-up frequency of use: n (%)**	**Non-daily**	0 (0)	35 (19)	0 (0)	62 (13.8)	
	**Daily**	229 (100)^b^	149 (81)	9 (100)	387 (86.2)	
**Intensity (g/week): median (IQR)**	**Baseline**	4.2 (5.6)^b^	1 (3.8)	2.8 (2.8)	2.4 (3.6)	
	**Follow-up**	4.2 (4.2)^b^	2.8 (4.9)	4.2 (5.6)	2.8 (5.6)	
**Recency of use (h): median (IQR)**		3 (3)^b^	11 (6)	9 (12)	4 (10)	

SD = standard deviation; IQR = interquartile range

a p-value<0.05 compared with participants who did not use opium.

b p-value<0.05 compared with opium use by smoking.

c Total number of participants who used opium at baseline is 447.

d Total number of participants who used opium stratified by follow-up opium type is 411 due to missing opium type data.

⁎ Number of “any use” is higher than sum of the presented routes due to missing route data.

A significant shift towards the ingestion route of opium use was observed in the follow-up visit compared with the baseline, as 39 % of those who used opium by smoking at baseline shifted to ingestion, whereas none of thosereporting ingestion of opium at baseline shifted to smoking (Table 1). At baseline, teriak was the most common opium type; however, follow-up data showed a more balanced distribution, with a slight preference for shireh in the ingestion group (57.8 % vs. 38.8 %).

**Fig. 1:**
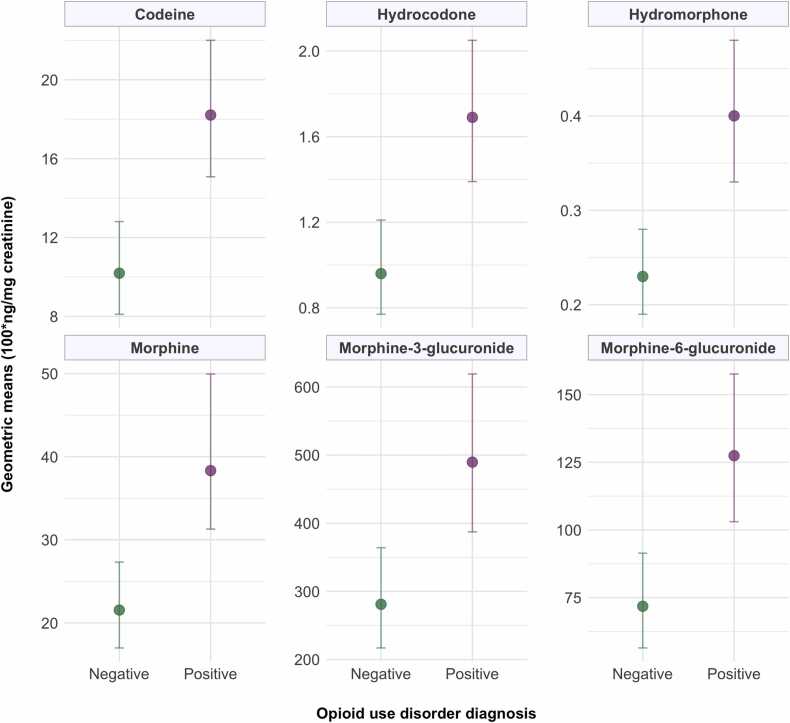
Geometric means (100*ng/mg creatinine) and 95% confidence intervals of opioid biomarker concentrations by opioid use disorder diagnosis, using samples of the follow-up assessment. Each panel visualizes the data corresponding to the specified opioid biomarkers, with the point estimates representing the geometric means and error bars representing the 95% confidence intervals.

As presented in Table 2 (creatinine-corrected concentrations) and Supplementary Table S3 (non-corrected concentrations), the follow-up assessment showed higher geometric means for most opioid biomarkers than the baseline. In the adjusted models, ingestion and daily use were consistently associated with higher concentrations of all opioid biomarkers, with the highest geometric means observed in association with ingestion at both time points. Shireh use was significantly associated with elevated concentrations of morphine at baseline and all biomarkers except codeine and morphine-3-glucuronide in the follow-up. A dose-response relationship was observed across quartiles of use intensity for all opioid biomarkers in both assessments, persisting after adjustment for route and type of opium. Having a diagnosis of OUD was also associated with significantly higher concentrations of all opioid biomarkers compared with not having an OUD diagnosis(Fig. 1). All associations in the follow-up models remained significant after adjustment for recency.

**Table 2 –:** Geometric means (100⁎ng/mg creatinine) of opioid biomarker concentrations by self-reported patterns of opium use at baseline and follow-up.

		**Geometric means (95 % confidence interval)**					
		**Codeine**	**Hydrocodone**	**Hydromorphone**	**Morphine**	**Morphine-** **3-glucuronide**	**Morphine-** **6-glucuronide**
**Baseline**							
**Total opium use (n = 447)**		14.2 (12.1–16.6)	0.9 (0.7–1.0)	0.2 (0.2–0.2)	20.8 (17.5–24.7)	232.5 (191.5–282.2)	57.1 (47.9–68.0)
**Route**^a^	**Ingestion (n = 109)**	38.5 (30.6–48.3)	3.7 (2.9–4.7)	0.8 (0.6–1.1)	65.2 (50.2–84.7)	903.9 (664.9–1228.8)	219.3 (167.1–287.8)
	**Smoking (n = 327)**	9.8 (8.2–11.8)⁎	0.5 (0.4–0.6)⁎	0.1 (0.1–0.1)⁎	13.6 (11.2–16.5)⁎	140.4 (113.1–174.3)⁎	34.5 (28.5–41.9)⁎
**Frequency**	**Non-daily (n = 159)**	5.1 (3.9–6.6)	0.3 (0.2–0.4)	0.1 (0.1–0.1)	6.4 (4.8–8.5)	68.0 (48.8–94.7)	17.3 (13.0 – 23.0)
	**Daily (n = 288)**	24.9 (21.2–29.4)⁎	1.5 (1.3–1.8)⁎	0.3 (0.3–0.4)⁎	39.7 (33.3–47.4)⁎	457.3 (374.0–559.2)⁎	110.1 (91.6–132.3)⁎
**Type**^a^	**Teriak (n = 386)**	12.4 (10.5–14.6)	0.7 (0.6–0.9)	0.2 (0.2–0.2)	17.5 (14.6–21.0)	195.8 (159.3–240.7)	47.9 (40.0–57.7)
	**Shireh (n = 52)**	34.9 (22.7–53.7)	2.1 (1.3–3.4)	0.5 (0.3–0.8)	64.1 (39.8–103.3)⁎	683.5 (393.4–1187.7)	176.0 (107.6–288.0)
**Intensity quartiles**^b^	**0.05–0.6 (n = 125)**	4.3 (3.2–5.8)	0.3 (0.2–0.4)	0.1 (0.1–0.1)	5.2 (3.7–7.1)	52.5 (35.7–77.2)	13.9 (10.0–19.3)
	**0.7–2.4 (n = 99)**	14.6 (10.7–19.9)†, §	0.8 (0.6–1.1)†, §	0.2 (0.1–0.3)†, §	22.6 (16.5–30.9)†, §	256.3 (186.1–352.8)†, §	60.4 (44.3–82.4)†, §
	**2.5–4.2 (n = 123)**	25.0 (19.5–32.1)†	1.5 (1.2–2.0)†	0.4 (0.3–0.5)†, §	42.1 (32.4–54.8)†	461.1 (337.3–630.4)†	114.5 (86.9–150.9)†
	**4.3–33.6 (n = 100)**	30.3 (23.3–39.4)	1.8 (1.4–2.4)	0.4 (0.3–0.6)	46.3 (34.4–62.1)	590.2 (417.9–833.6)	135.3 (99.3–184.5)
**Follow-up**							
**Total opium use (n = 449)**		14.3 (12.3–16.5)	1.3 (1.2–1.6)	0.3 (0.3–0.4)	30.1 (25.7–35.1)	387.4 (325.0–461.8)	100.0 (85.1–117.6)
**Route**^a^	**Ingestion (n = 229)**	31.2 (26.3–37.1)	4.0 (3.4–4.6)	0.8 (0.7–0.9)	71.9 (60.9–84.9)	1115.0 (951.5–1306.5)	272.0 (232.9–317.8)
	**Smoking (n = 184)**	7.3 (6.0–8.9)⁎	0.5 (0.4–0.5)⁎	0.1 (0.1–0.2)⁎	15.1 (12.3–18.6)⁎	182.2 (149.2–222.6)⁎	45.4 (37.1–55.4)⁎
**Frequency**	**Non-daily (n = 62)**	2.2 (1.6–3.1)	0.2 (0.2–0.3)	0.1 (0.1–0.1)	2.9 (2.0–4.3)	22.1 (12.5–39.2)	8.3 (5.3–13.0)
	**Daily (n = 387)**	19.2 (16.7–22.2)⁎	1.8 (1.5–2.1)⁎	0.4 (0.4–0.5)⁎	43.7 (38.1–50.2)⁎	613.5 (536.0–702.3)⁎	149.1 (130.0–171.0)⁎
**Type**^a^	**Teriak (n = 198)**	13.0 (10.4–16.2)	1.1 (0.8–1.3)	0.3 (0.2–0.3)	27.0 (21.4–34.1)	375.6 (293.5–480.6)	90.4 (71.2–114.8)
	**Shireh (n = 203)**	21.1 (17.4–25.6)	2.2 (1.8–2.7)⁎	0.5 (0.4–0.6)⁎	50.5 (42.3–60.4)⁎	686.9 (579.3–814.4)	172.5 (144.9–205.4)⁎
**Intensity quartiles**^b^	**0.1–1.4 (n = 121)**	8.6 (6.4–11.6)	0.7 (0.6 – 1.0)	0.2 (0.2–0.3)	17.3 (12.7–23.6)	228.3 (167.1–311.8)	57.0 (41.9–77.7)
	**1.5–2.8 (n = 87)**	25.5 (19.0–34.3)†	2.6 (1.9–3.4)†	0.5 (0.4–0.7)†	56.2 (42.6–74.2)†	723.5 (550.9–950.2)†	190.0 (144.6–249.5)†
	**2.9–7.0 (n = 113)**	18.0 (13.8–23.5)†	1.6 (1.2–2.1)†	0.4 (0.3–0.5)†	43.0 (33.1–55.9)†	642.1 (496.5–830.5)†	145.5 (111.9–189.1)†
	**7.1–42.0 (n = 90)**	25.3 (19.6–32.7)	2.5 (1.9–3.2)	0.6 (0.5–0.8)	61.4 (49.0–76.8)	891.0 (706.5–1123.6)	217.7 (173.3–273.4)

a Individuals who used both types or both routes of opium are not included and therefore the total number of individuals who used opium is higher than sum of both types or routes.

b Unit: grams per week.

⁎ p-value< 0.05 from multivariate linear regression models including all listed opium use characteristics as independent variables; for intensity, this shows p-for-trend from modeling intensity as an ordinal variable.

† p-value< 0.05 compared to the first quartile of intensity, based on modeling intensity as a categorical variable with the first quartile as the reference.

§ p-value< 0.05 compared to the fourth quartile of intensity, based on modeling intensity as a categorical variable with the fourth quartile as the reference.

**Fig. 2:**
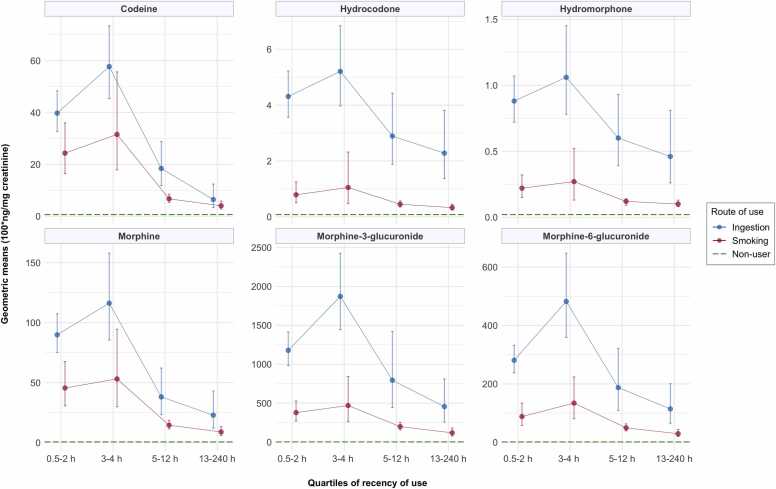
Geometric means (100*ng/mg creatinine) and 95% confidence intervals of opioid biomarker concentrations across quartiles of recency of use and stratified by route of opium use, using samples of the follow-up assessment. Each panel visualizes the data corresponding to the specified opioid biomarkers, with the point estimates showing the geometric means, and error bars representing the 95% confidence intervals of the opioid biomarker concentrations.

Opioid biomarker concentrations showed a clear time-dependent trend across quartiles of recency, stratified by route of opium use (p-values<0.05). Concentrations of all opioid biomarkers, regardless of the route of opium use, followed a similar pattern across quartiles of time since the most recent use (Fig. 2). Concentrations peaked when opium was used 3–4 h before sample collection and declined among those who had used opium 5 h or more before, but remained in the detectable range even after several days. The biomarker concentrations were also higher among individuals who used opium by ingestion in all recency quartiles compared with those who used by smoking.

We explored how the changes in the patterns of use impact the opioid biomarker concentrations at follow-up (Supplementary Table S4). Compared with continued smoking, both continued ingestion and transitioning from smoking to ingestion were associated with substantially increased opioid biomarker concentrations. Switching from teriak to shireh was associated with increased concentrations of hydrocodone and hydromorphone; however, transitioning to teriak or continued shireh use did not result in significant changes compared to continued teriak use. Non-daily use, whether continued or adopted after daily use, was associated with decreased opioid biomarker concentrations in comparison with continued daily use.

Opioid biomarker measurements had good reliability in the two urine samples taken over the 10-year interval, with ICCs ranging from 0.69 to 0.78 for all those who used opium. As detailed in Table 3, reliability remained high for almost all patterns of use, except among those who ingested opium (ICCs=0.36–0.60) and those who used opium on a non-daily basis (ICCs=0.17–0.64).

**Table 3 –:** Reliability of opioid biomarker measurements over a 10-year interval among individuals who used opium, stratified by use patterns.

	**Intraclass correlation coefficients (95 % confidence interval)**					
	**Codeine**	**Hydrocodone**	**Hydromorphone**	**Morphine**	**Morphine-** **3-glucuronide**	**Morphine-** **6-glucuronide**
**Total use (n = 447)**	0.72 (0.66–0.77)⁎	0.78 (0.74–0.82)⁎	0.78 (0.74–0.82)⁎	0.73 (0.68–0.78)⁎	0.69 (0.62–0.74)⁎	0.73 (0.68–0.78)⁎
**Non-OUD (n = 189)**	0.77 (0.70–0.83)⁎	0.82 (0.76–0.86)⁎	0.79 (0.73–0.85)⁎	0.81 (0.75–0.86)⁎	0.78 (0.70–0.83)⁎	0.82 (0.76–0.86)⁎
**OUD (n = 260)**	0.64 (0.55–0.72)⁎	0.73 (0.66–0.79)⁎	0.76 (0.69–0.81)⁎	0.63 (0.52–0.71)⁎	0.58 (0.46–0.67)⁎	0.63 (0.54–0.72)⁎
**Teriak (n = 187)**	0.77 (0.70–0.83)⁎	0.85 (0.80–0.89)⁎	0.85 (0.80–0.89)⁎	0.82 (0.75–0.86)⁎	0.79 (0.73–0.85)⁎	0.85 (0.79–0.88)⁎
**Shireh (n = 42)**	0.74 (0.51–0.86)⁎	0.74 (0.52–0.86)⁎	0.75 (0.55–0.87)⁎	0.74 (0.51–0.86)⁎	0.75 (0.54–0.87)⁎	0.77 (0.57–0.88)⁎
**Ingestion (n = 99)**	0.45 (0.18–0.63)⁎	0.48 (0.22–0.65)⁎	0.60 (0.40–0.73)⁎	0.44 (0.17–0.62)⁎	0.36 (0.04–0.57)⁎	0.42 (0.14–0.61)⁎
**Smoking (n = 183)**	0.66 (0.55–0.75)⁎	0.67 (0.55–0.72)⁎	0.66 (0.54–0.74)⁎	0.72 (0.62–0.79)⁎	0.67 (0.56–0.76)⁎	0.71 (0.61–0.78)⁎
**Non-daily use (n = 45)**	0.37 (−0.15–0.65)	0.64 (0.35–0.80)⁎	0.58 (0.23–0.77)⁎	0.41 (−0.07–0.68)⁎	0.17 (−0.51–0.54)	0.27 (−0.33–0.60)
**Daily use (n = 271)**	0.65 (0.56–0.73)⁎	0.73 (0.65–0.78)⁎	0.75 (0.68–0.80)⁎	0.66 (0.57–0.73)⁎	0.60 (0.49–0.68)⁎	0.68 (0.59–0.75)⁎

OUD=  opioid use disorder

⁎ p-value< 0.05 from F-test of the mixed-effects model.

## 4 Discussion

We found opioid biomarkers to be reliable reflections of subjective characterizations of opium use, with clear dose-response relationships relative to self-reported doses, and good reliability over a 10-year interval. Use by ingestion, daily use, and OUD diagnosis were associated with significantly higher biomarker concentrations compared to smoking, non-daily use, and absence of OUD diagnosis, respectively. Concentrations declined after peaking when opium was used 3–4 h before sample collection, but they remained in the detectable range even several days after use.

We measured six urinary opioids to capture the chemically complex nature of opium, which consists of several alkaloids, primarily morphine and codeine (Labanca et al., 2018, Lim and Kwok, 1981). Concentrations of these opioid biomarkers in our sample were generally higher than those reported in previous opioid use studies. In our follow-up assessment, the geometric mean of morphine was reported at 2.58 mg/L, morphine-3-glucuronide at 33.28 mg/L, morphine-6-glucuronide at 8.59 mg/L, and codeine at 1.22 mg/L (Supplementary Table S3). Previously, a case report documented that long-term ingestion of 1 g/day of opium dross resulted in urine concentrations of 0.64 mg/L for morphine and 0.37 mg/L for codeine (Cone et al., 1982). In the post-mortem urine analysis of 36 individuals who used heroin, morphine concentrations were reported at 4.28 mg/L, with 27.8 mg/L of morphine-3-glucuronide and 4.52 mg/L of morphine-6-glucuronide. Similarly, in 49 post-mortem urine samples of individuals who used morphine, concentrations of morphine, morphine-3-glucuronide, and morphine-6-glucuronide were 2.54, 31, and 4.69 mg/L, respectively (Jakobsson et al., 2020). In a study on 11 individuals given a single dose of heroin, peak urinary morphine concentrations ranged from 0.117 to 2.58 mg/L, with a median value of 1.4 mg/L following high doses (10.5–13.9 mg) (Smith et al., 2001). Although comparisons should be interpreted with caution due to differences in study designs, populations, and methods of use, our results show that those who used opium chronically in this population (about 17 % of the cohort), are exposed to relatively high levels of opioid alkaloids, highlighting the clinical and public health implications of this common long-term exposure.

Our previous study on this sample of individuals who used opium found significantly higher intensity and frequency of use among individuals with OUD (Alvand et al., 2023), consistent with prescription opioid use studies (Hser et al., 2019, Huffman et al., 2015). Higher intensity and frequency of use, whether in the context of OUD or as separate characteristics of use, naturally indicate a greater total opium intake, which has been found to correspond to increased opioid biomarker concentrations in hair and blood samples (Sabzevari et al., 2004, Somogyi et al., 2008). Our findings confirmed these associations for the spot urine opioid metabolites for the first time.

Biomarker concentrations remained in the detectable range across all recency quartiles, distinguishable from those who did not use opium regardless of time elapsed since last use. Recency of use, irrespective of the consumption route, impacted biomarker concentrations, with the highest concentrations observed among those who had last used opium 3–4 h before the urine test. Even though the pharmacokinetics of opium is understudied (IARC Working Group on the Identification of Carcinogenic Hazards to Humans, 2021), similar reports exist in the literature. The maximum concentrations of morphine and codeine after brown mixture consumption (a cold prescription drug containing opium powder and tincture) have been reported at 2–6 h after use (Liu et al., 2006). The maximum morphine concentrations after ingestion of a single dose of medicinal opium (containing 2.5 mg of morphine and a smaller amount of codeine) also occurred 2–4 h after use (Yong and Lik, 1977). While recency had a clear impact on biomarker concentrations, adjusting for recency in models examining types, routes, frequency, and intensity did not diminish the significance of the observed associations, further confirming that these biomarkers are appropriate biomarkers of long-term opium exposure.

We observed significantly higher concentrations of opioid biomarkers from ingestion compared with smoking. While smoking has been repeatedly identified as the most common route of opium use (IARC Working Group on the Identification of Carcinogenic Hazards to Humans, 2021, Kalant, 1997, Sheikh et al., 2020), we observed a significant shift to ingestion after a 10-year interval. Switching to opium ingestion from smoking was associated with increased opioid biomarker concentrations, even after adjustment for dose changes. The difference in drug absorption between the routes seems to be a key contributor to this observation. Ingested opium is fully absorbed into the bloodstream, but only a fraction of the smoked opium is absorbed, as some portions do not vaporize, and not all vaporized compounds reach the lungs (Kalant, 1997). The route of opium use also affects exposure to other toxicants, which have been proposed as potential contributors to opium carcinogenicity (IARC Working Group on the Identification of Carcinogenic Hazards to Humans, 2021, Vadhel et al., 2023); compared with smoking, opium ingestion is associated with lower exposure to polycyclic aromatic hydrocarbons (Naghash et al., 2025) but higher lead exposure (Etemadi et al., 2022). These findings further highlight the importance of considering the route of opium use in opioid risk assessment. In comparison with smoking, opium ingestion is associated with a higher risk of all-cause and gastrointestinal disease mortality and incidence of liver, brain, and head and neck cancers (Khademi et al., 2012, Malekzadeh et al., 2013, Mohebbi et al., 2021, Sheikh et al., 2020), and biomarkers can provide essential clues to uncovering the mechanism(s) for opium carcinogenicity.

Opioid biomarker concentrations remained consistent over a 10-year interval, confirming their reliability as markers of long-term exposure. The ICCs were very good for all six biomarkers in the total sample (0.69–0.78) and stratified by patterns of use (≥0.60), with only two exceptions. The ICCs were moderately decreased for the ingestion group despite similar intensity and frequency of use between the time points (p-values>0.05). This can be attributed to the inherent pharmacokinetic variability associated with gastrointestinal absorption mediated by food and medicine intake variations (Vinarov et al., 2021). Reliability was also lower among those who used opium non-daily compared with those who used opium daily, potentially due to fluctuations in the biomarker concentration caused by sporadic use patterns.

This is the first study to correlate a comprehensive panel of opioid biomarkers with detailed self-reported characteristics of opium use over a follow-up of about 10 years. Even though individual variations in metabolism and renal excretion could have influenced measured concentrations, the overall results were robust enough to show strong correlations with the characterizations of opium use. Given that this study was conducted among individuals who used opium from a single region in Iran, the generalizability of findings to the general population may be limited. Larger studies across different geographical areas with diverse demographics or usage habits are warranted to further validate these results. While we have examined and accounted for the changes in the characteristics of opium consumption, which are the primary determinants of opioid concentrations, unmeasured factors such as lifestyle or health changes may still have introduced residual confounding in our longitudinal assessments. While opium use is not stigmatized in this area, the possibility of misclassifications and biased self-reports cannot be ruled out.

## 5 Conclusion

In conclusion, opioid biomarkers (codeine, hydrocodone, hydromorphone, morphine, morphine-3-glucuronide, and morphine-6-glucuronide) are valid markers of opium use for a lifetime history of use, route, frequency, intensity, and recency of use and OUD diagnosis and show established consistency during a 10-year interval.

## Author disclosures

All authors confirm that this manuscript has not been published previously, is not currently under consideration for publication elsewhere and if accepted, will not be published elsewhere in any form. All authors have approved the manuscript and agreed to its submission to *Drug and Alcohol Dependence Reports* journal. The authors declare no competing interests.

## CRediT authorship contribution statement

**Arash Etemadi:** Writing – review & editing, Validation, Supervision, Methodology, Formal analysis, Data curation, Conceptualization. **Rebecca L. Shaner:** Writing – review & editing, Methodology, Data curation. **Reza Malekzadeh:** Writing – review & editing, Supervision, Resources, Project administration, Funding acquisition, Conceptualization. **Mahdokht Naghash:** Writing – review & editing, Writing – original draft, Visualization, Methodology, Formal analysis, Data curation. **Gholamreza Roshandel:** Writing – review & editing, Project administration, Data curation. **Hossein Poustchi:** Writing – review & editing, Resources, Project administration, Data curation. **Abraham Tuachi:** Writing – review & editing, Methodology, Data curation. **Katrice D. Williams:** Writing – review & editing, Methodology, Data curation. **Farin Kamangar:** Writing – review & editing, Validation, Supervision. **Christian C. Abnet:** Writing – review & editing, Supervision, Resources, Project administration, Funding acquisition. **Paolo Boffetta:** Writing – review & editing, Supervision, Project administration. **Neal D. Freedman:** Writing – review & editing, Supervision, Project administration, Funding acquisition. **Elizabeth I. Hamelin:** Writing – review & editing, Supervision, Methodology, Data curation.

## Funding

This study was supported by the Intramural Research Program of the National Cancer Institute, National Institutes of Health (N.D. Freedman, C.C. Abnet, A. Etemadi). The funders were not involved in the study design, data collection and analysis, decision to publish, or preparation of the manuscript.

## Disclaimer

The contributions of the NIH authors are considered Works of the United States Government. The findings and conclusions presented in this paper are those of the authors and do not necessarily reflect the views of the NIH, the Centers for Disease Control and Prevention (CDC) or the U.S. Department of Health and Human Services. Use of trade names is for identification only and does not imply endorsement by the CDC, the Public Health Service, or the U.S. Department of Health and Human Services.

## Declaration of Competing Interest

The authors declare that they have no known competing financial interests or personal relationships that could have appeared to influence the work reported in this paper.

## Data Availability

The de-identified data underlying this article can be shared upon reasonable request and approval through the study portal: https://dceg2.cancer.gov/gemshare
