# Supplementary material for: Opioid biomarkers in urine as reliable and valid correlates of opium use characteristics: A 10-year longitudinal assessment
Source: Drug Alcohol Depend Rep. 2025 Aug 30;17:100377. doi: 10.1016/j.dadr.2025.100377 (PMC12451287; doi:10.1016/j.dadr.2025.100377)
Supplement: Supplementary file 1 — Supplementary material [file mmc1.pdf]

Supplementary Material for Manuscript Entitled “**Opioid Biomarkers in Urine as Reliable and Valid Correlates of Opium Use Characteristics: A 10-year Longitudinal Assessment”**

Supplementary Table S1 – Mass spectrometric parameters for each analyte

**Analyte**	**Parameter**					
	**Precursor Ion (*m/z*)**	**Product Ion (*m/z*)**	**Declustering Potential (V)**	**Entrance Potential (V)**	**Collision Energy (eV)**	**Cell Exit Potential (V)**
**Codeine**	299.8	215.0*	144	5	35	18
		165.0	144	5	53	15
	305.9	218.1†	125	7	37	28
**Hydrocodone**	299.9	198.9*	137	8	39	25
		171.2	137	8	50	21
	305.9	202.0†	145	10	46	24
**Hydromorphone**	285.8	184.9*	157	6	40	23
		156.9	157	6	55	20
	288.8	184.9†	161	9	40	18
**Morphine**	285.9	201.0*	138	11	34	24
		165.0	138	11	52	14
	288.9	201.0†	160	6	36	17
**Morphine-3-glucuronide**	462.0	201.0*	180	4	59	19
		165.0	180	4	85	14
	465.1	201.0†	42	5	56	17
**Morphine-6-glucuronide**	462.0	201.2*	156	6	54	11
		165.0	156	6	71	22
	473.0	211.2†	145	10	46	24

Transitions with a * were used for quantitation, a † indicates an internal standard transition.

Supplementary Table S2 – Coefficients of variation and accuracy of the quality control material for opioid biomarkers

	QC Low		QC Med		QC High		Blinded spikes
	Accuracy (%)	CV (%)	Accuracy (%)	CV (%)	Accuracy (%)	CV (%)	CV (%)
**Codeine**	106.6	5.52	103.5	3.71	94.2	5.86	18.26
**Hydrocodone**	101.0	4.21	99.4	4.03	97.6	5.71	17.10
**Hydromorphone**	93.6	5.16	97.9	4.97	101.9	7.25	20.74
**Morphine**	100.4	3.86	99.3	2.52	97.9	6.01	25.76
**Morphine-3-glucuronide**	101.3	4.45	96.7	3.22	102.1	3.91	13.30
**Morphine-6-glucuronide**	97.8	7.33	99.1	8.28	99.4	7.61	18.72

QC=quality control; CV=coefficient of variation

Supplementary Table S3 – Geometric means (mg/L) of opioid biomarker concentrations by self-reported patterns of opium use at baseline and follow-up

		Geometric means (95% confidence interval)					
		**Codeine**	**Hydrocodone**	**Hydromorphone**	**Morphine**	**Morphine-3-glucuronide**	**Morphine-6-glucuronide**
**Baseline**							
**Total opium use (n=447)**		1.50 (1.28-1.74)	0.09 (0.08-0.10)	0.02 (0.02-0.02)	2.19 (1.86-2.59)	24.55 (20.29-29.69)	6.02 (5.08-7.15)
**Route[a]**	**Ingestion (n=109)**	3.41 (2.62-4.43)	0.33 (0.25-0.43)	0.07 (0.05-0.10)	5.78 (4.32-7.73)	80.11 (57.50-111.63)	19.44 (14.44-26.16)
	**Smoking (n=327)**	1.10 (0.92-1.31)*	0.06 (0.05-0.06)*	0.01 (0.01-0.02)*	1.52 (1.26-1.84)*	15.72 (12.71-19.44)*	3.87 (3.21-4.66)*
**Frequency**	**Non-daily (n=159)**	0.66 (0.50-0.86)	0.04 (0.03-0.05)	0.01 (0.01-0.01)	0.83 (0.62-1.10)	8.80 (6.29-12.30)	2.24 (1.67-2.99)
	**Daily (n=288)**	2.36 (2.00-2.78)*	0.14 (0.12-0.17)*	0.03 (0.03-0.04)*	3.75 (3.13-4.49)*	43.17 (35.21-52.95)*	10.39 (8.65-12.50)*
**Type[a]**	**Teriak (n=386)**	1.31 (1.12-1.55)	0.08 (0.07-0.09)	0.02 (0.02-0.02)	1.86 (1.56-2.22)	20.82 (17.01-25.49)	5.09 (4.25-6.10)
	**Shireh (n=52)**	3.66 (2.38-5.63)	0.22 (0.13-0.37)	0.05 (0.03-0.08)	6.73 (4.18-10.83)*	71.72 (41.01-125.44)	18.47 (11.25-30.32)
**Intensity quartiles[b]**	**0.05-0.6 (n=125)**	0.56 (0.42-0.76)	0.04 (0.03-0.05)	0.01 (0.01-0.01)	0.67 (0.48-0.93)	6.81 (4.61-10.07)	1.80 (1.29-2.52)
	**0.7-2.4 (n=99)**	1.64 (1.20-2.23)†§	0.09 (0.07-0.12)†§	0.02 (0.02-0.03)†§	2.53 (1.86-3.45)†§	28.77 (21.04-39.34)†§	6.78 (5.01-9.18)†§
	**2.5-4.2 (n=123)**	2.12 (1.66-2.71)†	0.13 (0.10-0.17)†	0.03 (0.02-0.04)†§	3.57 (2.74-4.64)†	39.05 (28.58-53.37)†	9.70 (7.38-12.74)†
	**4.3-33.6 (n=100)**	3.05 (2.30-4.05)†*	0.18 (0.14-0.25)†*	0.04 (0.03-0.06)†*	4.66 (3.39-6.40)†*	59.46 (41.31-85.58)†*	13.63 (9.85-18.86)†*
**Follow-up**							
**Total opium use (n=449)**		1.22 (1.06-1.41)	0.11 (0.10-0.13)	0.03 (0.02-0.03)	2.58 (2.21-3.02)	33.28 (27.94-39.63)	8.59 (7.32-10.09)
**Route[a]**	**Ingestion (n=229)**	2.34 (1.95-2.80)	0.30 (0.25-0.35)	0.06 (0.05-0.07)	5.38 (4.48-6.46)	83.45 (69.64-100.01)	20.36 (17.10-24.25)
	**Smoking (n=184)**	0.73 (0.60-0.89)*	0.05 (0.04-0.05)*	0.01 (0.01-0.01)*	1.52 (1.24-1.87)*	18.33 (14.97-22.43)*	4.56 (3.72-5.59)*
**Frequency**	**Non-daily (n=62)**	0.23 (0.16-0.32)	0.02 (0.02-0.03)	0.01 (0.00-0.01)	0.30 (0.20-0.46)	2.28 (1.27-4.10)	0.86 (0.54-1.37)
	**Daily (n=387)**	1.60 (1.39-1.85)*	0.15 (0.13-0.17)*	0.03 (0.03-0.04)*	3.65 (3.17-4.20)*	51.18 (44.55-58.80)*	12.44 (10.82-14.30)*
**Type[a]**	**Teriak (n=198)**	0.66 (0.50-0.86)	0.04 (0.03-0.05)	0.01 (0.01-0.01)	0.83 (0.62-1.10)	8.80 (6.29-12.30)	2.24 (1.67-2.99)
	**Shireh (n=203)**	2.36 (2.00-2.78)	0.14 (0.12-0.17)*	0.03 (0.03-0.04)*	3.75 (3.13-4.49)*	43.17 (35.21-52.95)	10.39 (8.65-12.50)*
**Intensity quartiles[b]**	**0.1-1.4 (n=121)**	0.79 (0.60-1.04)	0.07 (0.05-0.09)	0.02 (0.01-0.02)	1.59 (1.19-2.14)	20.99 (15.62-28.20)	5.24 (3.92-7.02)
	**1.5-2.8 (n=87)**	1.81 (1.36-2.42)†	0.18 (0.13-0.25)†	0.04 (0.03-0.05)†	3.99 (2.97-5.37)†	51.41 (37.26-70.93)†	13.50 (9.98-18.25)†
	**2.9-7.0 (n=113)**	1.46 (1.11-1.92)†	0.13 (0.10-0.17)†	0.03 (0.02-0.04)†	3.48 (2.64-4.59)†	52.00 (40.21-67.24)†	11.78 (8.95-15.52)†
	**7.1-42.0 (n=90)**	2.38 (1.81-3.12)†*	0.23 (0.17-0.30)†*	0.05 (0.04-0.07)†*	5.75 (4.48-7.39)†*	83.54 (65.20-107.05)†*	20.41 (16.12-25.84)†*

[a]Dual users of both types or both routes of opium are not included and therefore the number of total users is higher than sum of both types or routes.
[b]Unit: grams per week.
*p-value<0.05 from multivariate linear regression models on creatinine-corrected biomarker concentrations, including all listed opium use characteristics as independent variables; for intensity, this shows p-for-trend from modeling intensity as an ordinal variable.
†p-value<0.05 compared to the first quartile of intensity, based on modeling intensity as a categorical variable with the first quartile as the reference.
§p-value<0.05 compared to the fourth quartile of intensity, based on modeling intensity as a categorical variable with the fourth quartile as the reference.

Supplementary Table S4 – Associations between changes in patterns of opium use with follow-up opioid biomarker concentrations

	Linear regression exponentiated coefficients (95% confidence interval) for follow-up opioid biomarker concentrations[a]					
	**Codeine**	**Hydrocodone**	**Hydromorphone**	**Morphine**	**Morphine-3-glucuronide**	**Morphine-6-glucuronide**
**Continued smoking (n=183)**	Reference	Reference	Reference	Reference	Reference	Reference
**Switch to ingestion (n=119)**	1.81** (1.34-2.46)	4.48** (3.36-5.98)	3.09** (2.42-3.95)	1.88** (1.44-2.46)	2.65** (2.09-3.36)	2.37** (1.88-2.99)
**Continued ingestion (n=99)**	2.03** (1.42-2.92)	3.80** (2.58-5.58)	2.94** (2.08-4.16)	2.24** (1.59-3.15)	2.82** (1.92-4.13)	2.50** (1.74-3.59)
**Continued teriak (n=187)**	Reference	Reference	Reference	Reference	Reference	Reference
**Switch to shireh (n=157)**	1.13 (0.87-1.45)	1.59** (1.24-2.03)	1.33* (1.07-1.67)	1.23 (0.98-1.54)	1.13 (0.90-1.40)	1.23 (1.00-1.53)
**Continued shireh (n=42)**	1.16 (0.79-1.71)	1.40 (0.96-2.03)	1.40 (1.00-1.95)	1.21 (0.84-1.74)	1.21 (0.82-1.79)	1.22 (0.83-1.78)
**Switch to teriak (n=6)**	0.57 (0.22-1.50)	1.73 (0.77-3.87)	1.13 (0.56-2.29)	0.60 (0.27-1.34)	0.98 (0.47-2.01)	0.94 (0.48-1.86)
**Continued daily (n=271)**	Reference	Reference	Reference	Reference	Reference	Reference
**Switch to non-daily (n=17)**	0.53 (0.29-1.00)	0.27** (0.18-0.39)	0.30** (0.19-0.48)	0.32** (0.15-0.69)	0.22** (0.11-0.43)	0.30** (0.16-0.58)
**Continued non-daily (n=45)**	0.52* (0.28-0.96)	0.43** (0.26-0.71)	0.52** (0.33-0.82)	0.40** (0.22-0.74)	0.42* (0.21-0.83)	0.52* (0.28-1.00)
**Switch to daily (n=114)**	0.96 (0.71-1.30)	0.87 (0.64-1.18)	0.82 (0.62-1.07)	0.90 (0.69-1.18)	0.85 (0.65-1.11)	0.92 (0.71-1.20)

[a]Models were adjusted for sex, age, baseline opioid biomarker concentrations, recency of use and changes in intensity of use.
*P<0.05, **P<0.01
